# Preoperative 15 mg of melatonin for reducing anxiety and post-traumatic stress disorder symptoms in mandibular third molar surgery: A randomized double-blind placebo-controlled clinical trial

**DOI:** 10.4317/medoral.27846

**Published:** 2026-01-24

**Authors:** Helen Heloene Rosa, Carolina Ruppel, Nicolly da Silva Trappel, Ramon Cesar Godoy Goncalves, Marcelo Carlos Bortoluzzi

**Affiliations:** 1 Dentistry State University of Ponta Grossa (UEPG), Ponta Grossa, Brazil; 2 University Hospital of Campos Gerais (HUCG) Ponta Grossa, Brazil

## Abstract

**Background:**

This study evaluated the efficacy of a single 15 mg preoperative sublingual dose of melatonin in reducing anxiety as the primary outcome, and its effects on psychomotor performance, postoperative sleep quality, and early post-traumatic stress symptoms as secondary outcomes in patients undergoing mandibular third molar surgery.

**Material and Methods:**

Forty-eight patients were randomly allocated to receive either melatonin (n=24) or an identical placebo (n=24) sublingually 45 minutes before surgery. Anxiety was assessed using the State-Trait Anxiety Inventory (STAI) and a Visual Analog Scale for Anxiety (VAS-A) at baseline, 45 minutes post-medication, and post-surgery. Intraoperative anxiety was measured with the surgeon-rated Interval Scale of Anxiety Response (ISAR). Psychomotor performance was evaluated with the Digit Symbol Substitution Test (DSST) at baseline and 45 minutes post-medication. Sleep quality was recorded via a patient diary for three postoperative nights, and post-traumatic stress symptoms were screened one week post-surgery using the Impact of Event Scale-Revised (IES-R).

**Results:**

After adjusting for baseline scores using ANCOVA and GLM, no significant effect of melatonin was observed on state anxiety at any time point. Regarding secondary outcomes, no significant differences were observed in psychomotor performance, intraoperative anxiety from the surgeon's perspective (ISAR), postoperative sleep quality across the three nights, or early post-traumatic stress symptoms. The intervention was well tolerated, with no adverse events reported.

**Conclusions:**

A single 15 mg preoperative dose of sublingual melatonin did not demonstrate any significant benefit over placebo in reducing perioperative anxiety, improving psychomotor performance, enhancing postoperative sleep quality, or preventing early post-traumatic stress symptoms in patients undergoing mandibular third molar surgery.

## Introduction

Preoperative anxiety, often referred to as dental anxiety or fear, is a well-recognized psychological stressor in third molar removal, one of the most frequent oral and maxillofacial procedures (1 , 2). It is marked by apprehension, hypervigilance, and physiological arousal, with pain anticipation during and after surgery as a central concern (1 - 4). Elevated preoperative anxiety is consistently linked to worse outcomes, particularly increased postoperative pain (2 , 5), and can intensify intraoperative discomfort (2). Moreover, this combination of anxiety and discomfort may render the procedure more traumatic, raising the risk of Post-Traumatic Stress Disorder (PTSD) symptoms in susceptible patients (1).

Melatonin (N-acetyl-5-methoxytryptamine) is an indoleamine neurohormone primarily synthesized by the pineal gland, although extra-pineal production occurs in multiple tissues, including the retina, gastrointestinal tract, gingiva, and salivary glands (6 - 15). Its primary physiological role is the regulation of circadian rhythms and the sleep-wake cycle (6 , 9 , 11 - 16). In addition, melatonin exerts a wide range of biological effects, including antioxidant (6 - 9 , 12 - 15 , 17), anti-inflammatory (7 - 9 , 12 , 14 , 15), immunomodulatory (7 , 12 , 14 , 15), analgesic (7 , 9 , 12 , 13), and osteogenic properties (6 - 9 , 12 , 14 , 17).

In recent years, melatonin has been investigated as an alternative anxiolytic in the perioperative setting (8 , 10 , 12 , 13 , 16 , 18). Clinical studies in adults have reported that melatonin reduces preoperative anxiety when compared with placebo (10 , 12 , 16 , 19) and can provide anxiolysis comparable to that of benzodiazepines, such as midazolam and alprazolam (10 , 12 , 19 , 20). Unlike benzodiazepines, melatonin is generally associated with less sedation and minimal impairment of cognitive or psychomotor performance (10 , 12 , 16 , 19). Although a randomized trial in children found melatonin to be less effective than midazolam for reducing preoperative anxiety, its favorable safety profile remains a relevant advantage, particularly in light of the risks of respiratory suppression, paradoxical reactions, and postoperative agitation associated with benzodiazepines (18).

Given the established association between preoperative anxiety and adverse clinical and psychological outcomes, together with the growing evidence supporting melatonin as a safe anxiolytic agent, its potential role in oral and maxillofacial surgery warrants further investigation. The present study was designed to evaluate the efficacy of a single 15 mg preoperative sublingual dose of melatonin in patients undergoing mandibular third molar surgery. The primary outcome was the state anxiety, assessed 45 minutes after baseline measurement and soon after the surgery. Secondary outcomes included psychomotor performance, postoperative sleep quality, and early PTSD-related symptoms. It was hypothesized that, compared with placebo, melatonin would significantly reduce preoperative state anxiety and demonstrate superiority across the secondary outcomes, including psychomotor function, sleep quality, and post-traumatic stress response.

## Material and Methods

This randomized, double-blind, placebo-controlled clinical trial was conducted at an University Specialized Dental Surgery Clinic. The study protocol was prospectively registered in the Brazilian Registry of Clinical Trials (ReBEC; RBR-7h4zchf) prior to participant enrollment and received approval from the Institutional Ethics Committee (Approval Number: 6.850.728). All procedures were performed in strict accordance with the ethical principles of the Declaration of Helsinki. Written informed consent was obtained from all individual participants included in the study.

Participant selection

Participant recruitment was conducted between February 2024 and August 2025, enrolling individuals referred for surgical removal of a mandibular third molar at the dental school clinic.

Inclusion criteria required patients to: Be between 18 and 25 years of age; be classified as American Society of Anesthesiologists (ASA) physical status I or II; require surgical removal of a single impacted mandibular third molar; present no clinical signs of acute infection at the surgical site; not be using medications that affect the central nervous system (e.g., anxiolytics, antidepressants, antipsychotics, or mood stabilizers); have a Kharma scale classification ranging from easy to moderately difficult; and provide written informed consent to participate in the study.

Exclusion criteria included: A surgical time exceeding 50 minutes; loss to follow-up; noncooperative behavior; failure to complete postoperative assessments (e.g., diaries); and the occurrence of significant intraoperative complications (e.g., retained root fragments, clinically relevant alveolar hemorrhage).

Medication preparation, blinding, allocation and administration

Medication preparation, blinding, and allocation concealment were performed by an independent third party. Both the active drug (15 mg melatonin) and the placebo were manufactured as identical sublingual tablets, matching in appearance, taste, texture, and weight. The sublingual formulation was selected to ensure rapid absorption and avoid first-pass metabolism while maintaining blinding integrity. Tablets were packaged in identical opaque containers labeled only with random codes ("Medication A" or "Medication B"). The randomization key was securely held by the study administrator and remained inaccessible to investigators and participants until final data analysis.

Randomization followed a simple raffle procedure to achieve a 1:1 allocation ratio. Each participant selected a sealed, opaque envelope containing the assigned medication. A fixed 15 mg dose was used to enhance clinical applicability, reduce dosing variability, and reflect real-world use. Participants received a single tablet administered sublingually, ensuring that neither patients nor clinical or assessment staff could distinguish between melatonin and placebo.

Study instruments and questionnaires

Anxiety

The State-Trait Anxiety Inventory (STAI) is a questionnaire designed to evaluate an individual's anxiety level. The inventory consists of 40 questions divided into two subscales: State anxiety (STAI-S) and trait anxiety (STAI-T). The STAI-S reflects a temporary emotional state characterized by feelings of worry, whereas the STAI-T indicates a persistent tendency to perceive situations as threatening. Each category comprises 20 items, with scores ranging from 20 to 80, with higher scores indicating elevated levels of anxiety (21).

Visual Analog Scales (VAS) are simple tools used to measure subjective experiences, such as anxiety (VAS-A), on a continuum. They typically consist of a horizontal line ranging from "no sensation" at one end to "worst imaginable sensation" at the other end. Patients marked a point along the line that best reflected their current state. VAS is widely used in clinical settings due to its ease of use and ability to capture subtle changes in a patient's perceived intensity of symptoms (19).

Intraoperative anxiety was assessed immediately after surgery using the adapted Interval Scale of Anxiety Response (ISAR) (22). The scale comprises 11 surgeon-rated items, each scored from 0 (no sign) to 10 (very evident sign), evaluating: Physical signs (excessive perspiration, increased muscle tension, accelerated respiration, trembling); facial expressions (grimacing, wide-open eyes, forced eye closure, pallor); vocal cues (crying, moaning, shouting, sighing, voice tremor); self-reported fear; being anxious or nervous; procedure-related questioning (such as whether it would hurt, or whether it was truly necessary); interruptions during surgery; overall perceived anxiety and the extent to which anxiety interfered with the procedure. Additional items record signs of nausea or gag reflex as well as fainting. The final score is the sum of all 11 items.

Psychomotor function

The psychomotor function of patients was assessed using the Digit Symbol Substitution Test (DSST). The DSST is a validated paper-and-pencil evaluation of psychomotor speed, attention, and executive function. In this test, participants are presented with a key pairing the numbers 1-9 with unique abstract symbols. Below the key, rows of randomly ordered numbers are displayed, each followed by an empty box. The participant is instructed to fill in as many corresponding symbols as possible within a 90-second time limit, proceeding sequentially from left to right and top to bottom. The total score is the number of correct symbols substituted within the allotted time. A decrease in score is indicative of psychomotor impairment or cognitive dysfunction. The DSST was administered preoperatively (baseline) and again 45 minutes after study drug administration to evaluate changes in performance (16 , 19).

Sleep quality

In the patient diary, sleep quality on the first, second, and third postoperative nights was evaluated with a single-item question. Patients were asked to indicate their sleep experience by choosing from the following categories: "I slept well," "I had difficulty sleeping," or "I could not sleep" (23).

Post-traumatic stress disorder

The Impact of Event Scale-Revised (IES-R) was employed to screen for post-traumatic stress disorder (PTSD) symptomatology due to its strong discriminant validity. The IES-R comprises 22 items with five response options (never, a little, moderately, frequently, extremely), grouped into three subscales: Intrusion (reliving traumatic events), avoidance (efforts to avoid traumatic memories), and hyperarousal (increased excitability, including irritability, sleep difficulty, and hypervigilance). Higher scores indicate greater distress: 24 suggests clinical concern, 33 indicates probable PTSD, and 37 may reflect distress affecting immune function (1 , 24 , 25). In this study, the IES-R was administered during the one-week postoperative follow-up to capture the immediate psychological impact of surgery.

Dental surgery and operators

Two oral and maxillofacial surgeons, each with two years of post-residency experience and a similar surgical background, performed all extractions.

Study steps

**Figure 1:**
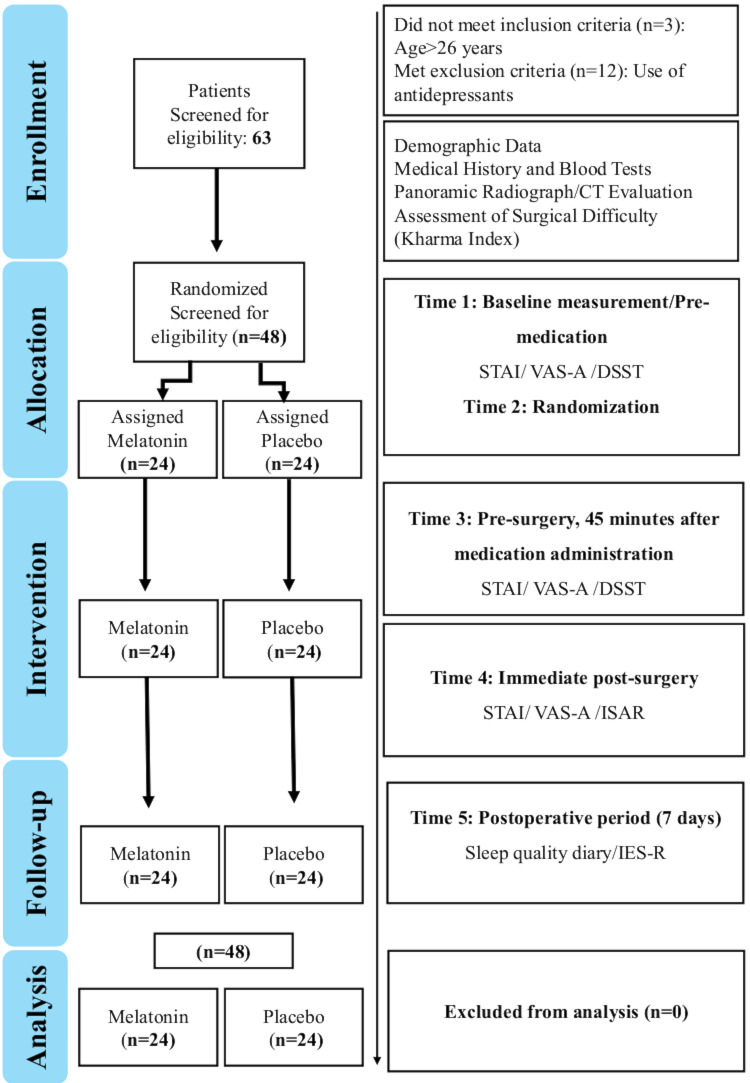
Flowchart of participant enrollment, allocation, intervention, follow-up, and analysis throughout the clinical trial.

Following enrollment and the provision of informed consent, eligible participants were randomly allocated to receive either a single 15 mg sublingual melatonin tablet or an identical placebo 45 minutes before surgery. Baseline assessments of anxiety and psychomotor function were conducted prior to medication administration and repeated 45 minutes afterward, immediately before the start of surgery. Intraoperative anxiety was rated by the surgeon upon completion of the procedure. Postoperative follow-up included a sleep quality diary completed over three consecutive nights and an assessment of post-traumatic stress symptoms conducted one week later. Protocol adherence and adverse events were monitored throughout the study period. A detailed summary of participant progression through the trial is provided in Figure 1.

1

Sample size

The sample size was calculated a priori using G*Power software (version 3.1). For the primary analysis, a repeated-measures ANOVA with a within-between interaction was selected. The calculation was based on an anticipated medium effect size (f = 0.25), an alpha error probability of 0.05, and a desired statistical power of 0.95. With two groups (melatonin and placebo) and three repeated measurements (baseline; preoperative, 45 minutes after the medication intervention and before the surgery to start and; immediate postoperative), a correlation among repeated measures of 0.5 was assumed. The calculation yielded a total required sample size of 44 participants (22 per group).

Statistical methods and procedures

Data were analyzed using Jamovi (https://www.jamovi.org/; version 2.7.6). Descriptive statistics were presented as means and standard deviations for continuous variables and as frequencies for categorical or nominal variables. Group comparisons at baseline were performed using the Chi-square test for categorical variables, Student's t-test for normally distributed continuous variables, and the Mann-Whitney U test for non-normally distributed variables.

Although the sample size calculation was based on the assumption of repeated-measures ANOVA for anxiety outcomes, significant baseline imbalances in the primary outcome were identified after data collection. Consequently, the pre-specified RM-ANOVA was not appropriate, as its validity relies on the assumption of baseline equivalence. The primary analysis was therefore changed to an analysis of covariance (ANCOVA) for the 45-minute post-medication measurement, controlling for the baseline score. The robustness of this finding was confirmed through a sensitivity analysis using a general linear model (GLM), which incorporated the baseline as a covariate and accounted for repeated measures.

Between-group comparisons for ISAR and IES-R scores were performed using the Mann-Whitney U test, while sleep quality responses were compared using Chi-square tests. Normality was assessed with the Shapiro-Wilk test and homogeneity of variances with Levene's test. All analyses were two-tailed, with statistical significance set at p&lt;0.05.

## Results

Sample characteristics

The final sample consisted of 48 patients undergoing mandibular third molar extraction surgery, the majority of whom were female (37 patients, 77%). Age ranged from 18 to 26 years, with a mean age of 21 years. There were no statistically significant differences in the distribution of gender (Chi-square test, p=0.7), age (Student's t test, p=0.7), or body mass index (Mann-Whitney U test, p=0.9) between the placebo and melatonin groups.

Surgical characteristics

According to the Kharma classification criteria for surgical difficulty, seven procedures were classified as easy, 33 as mild difficulty, and eight as moderate difficulty. No significant differences were observed among the surgical/medication groups considering Kharma classification criteria for surgical difficulty (Chi-square, p=0.7). Additional information about the tooth position can be seen in Table 1. Surgical duration was comparable between the intervention groups (Mann-Whitney U test, p=0.6), with a mean operative time of 27 minutes in the melatonin group and 25 minutes in the placebo group. Similarly, the volume of local anesthetic administered did not differ significantly between groups (Mann-Whitney U test, p=0.6).

Table 1

Dental anxiety

**Figure 2:**
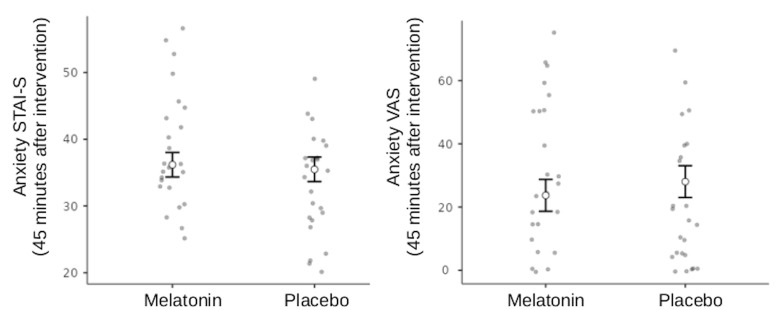
Adjusted preoperative state anxiety scores 45 minutes after sublingual administration of 15 mg melatonin or placebo. Bars represent estimated marginal means for both the State-Trait Anxiety Inventory-State (STAI-S, left) and the Visual Analog Scale for Anxiety (VAS-A, right), with adjustments for baseline scores via analysis of covariance (ANCOVA). No statistically significant differences between groups were observed for either STAI-S (p=0.6) or VAS-A (p=0.2).

Anxiety measured by the STAI showed a significant and uneven baseline between the medication groups (Student's t-test for STAI-State, Measure 1, melatonin mean 41.6, placebo mean 36.2, p=0.03). To compensate for this unbalanced baseline effect, an analysis of covariance (ANCOVA) was performed for the subsequent anxiety measures (measures 2 and 3), while controlling for the initial measure. The results showed no significant effect of melatonin on anxiety 45 minutes after medication intake (ANCOVA for STAI-State, Measure 2 controlled by Measure 1; p=0.6; ²=-0.004; ²=0.002). The estimated marginal mean after correction was 36.2 (95% CI, 34.3-38.0) for the melatonin group and 35.5 (95% CI, 33.7-37.3) for the placebo group (Figure 2).

2

The null finding was confirmed by a sensitivity analysis using a general linear model (GLM), which showed no significant effect of treatment on the post-medication score after adjusting for baseline (p = 0.60). Similarly, after surgery, no significant effect of melatonin on anxiety was observed (ANCOVA for STAI-State, Measure 3 controlled by Measure 1; p=0.6; ²=-0.008; ²=0.003). The estimated marginal means were 36.1 (95% CI, 33.4-38.7) for melatonin and 35.0 (95% CI, 32.4-37.7) for placebo. An additional analysis of postoperative anxiety was conducted using a general linear model (GLM) that included both the baseline and 45-minute scores as covariates. This model also indicated no significant treatment effect (p=0.63).

Similar to the STAI findings, the VAS-A scale revealed a significant baseline difference between groups (Student's t-test for VAS-A, Measure 1: Melatonin mean =42.8, placebo mean =22.4, p=0.01). Using the same statistical approach as for the STAI, the ANCOVA indicated that melatonin was not effective in reducing surgical anxiety (VAS-A, Measure 2 controlled by Measure 1; p=0.2; ²=-0.003; ²=0.009). The estimated marginal means after correction were 23.7 (95% CI, 18.7-28.7) for the melatonin group and 28.0 (95% CI, 23.0-33.1) for the placebo group. During the postoperative period, melatonin also showed no superiority over placebo (VAS-A, Measure 3 controlled by Measure 1; p=0.6), with estimated marginal means of 17.4 (95% CI, 9.5-25.2) for melatonin and 15.0 (95% CI, 7.2-22.8) for placebo. The raw descriptive values for STAI and VAS-A measures are presented in Table 2.

Table 2

Interval Scale of Anxiety Response (ISAR)

Intraoperative anxiety, was assessed by the surgeon immediately after surgery using the adapted Interval Scale of Anxiety Response (ISAR), showed no differences between groups (Mann-Whitney U test, p=0.63). The surgeon-rated descriptive values of ISAR responses are described in Table 3. During the procedure, no patients exhibited signs of nausea or gag reflex. However, one patient in the placebo group experienced a fainting episode.

Table 3

Psychomotor assessment

Psychomotor function, as assessed by the Digit Symbol Substitution Test (DSST), showed a significant interaction between treatment and time. At baseline, the mean DSST score was lower in melatonin group (54.1±9.5) compared to placebo group (59.7±10.5), a difference that approached but did not reach statistical significance (Student t test, p=0.060). Following drug administration, performance improved in both groups. However, the improvement was significantly greater in placebo group, which achieved a mean score of 67.1 (±11.8) measured 45 minutes post-administration and before surgery, compared to a mean score of 58.8 (±9.8) in melatonin group (Student t test, p=0.010).

Given the baseline imbalance in DSST scores, the analysis of the scores taken 45 minutes post-administration (and before surgery) was adjusted using ANCOVA, with the baseline score as a covariate. After this adjustment, the difference between groups was no longer statistically significant (ANCOVA, p=0.08), indicating that the treatment had no significant effect on psychomotor performance, accounting for the initial baseline difference.

Sleep quality

Postoperative sleep quality was assessed via patient diary for the first three nights following surgery. The distribution of responses regarding sleep experience;"slept well," "had difficulty sleeping," or "could not sleep"; was comparable between the melatonin and placebo groups across all nights. On the first night, the majority of participants in both groups reported sleeping well (70.8% melatonin, 66.7% placebo), with no statistically significant difference in the overall distribution of responses (p=0.51). This pattern remained consistent on the second (p=0.54) and third (p=0.36) nights, with over 75% of participants in each group reporting satisfactory sleep. Statistical analysis confirmed that preoperative melatonin administration did not result in a significant improvement in subjective sleep quality compared to the placebo on any of the three postoperative nights.

Post-traumatic stress disorder

Post-traumatic stress symptoms were assessed using the Impact of Event Scale-Revised (IES-R), with results summarized in Table 4. According to the established IES-R criteria, only two patients presented scores indicative of PTSD-related concerns (4%). One patient in the melatonin group scored 26 points, which is classified as representing a "clinical concern". A second patient, in the placebo group, scored 44 points, a level of distress associated with potential suppression of immune system functioning.

Table 4

Side effects

The intervention was well-tolerated. No adverse events were attributed to either melatonin or placebo administration throughout the study.

## Discussion

This randomized, double-blind, placebo-controlled clinical trial investigated the effect of a 15 mg sublingual single-dose melatonin on perioperative anxiety, psychomotor performance, postoperative sleep quality, and early PTSD-related symptoms in patients undergoing mandibular third molar extraction. Contrary to the initial hypothesis, melatonin did not demonstrate superior outcomes compared with placebo across the assessed domains.

Melatonin has been investigated in third molar surgery mainly for its anxiolytic, anti-inflammatory, and analgesic properties. Oral premedication with melatonin has demonstrated anxiolytic effects comparable to those of benzodiazepines such as midazolam and alprazolam, while offering the advantage of less sedation and preservation of psychomotor and cognitive function. This favorable profile has translated into higher patient and surgeon satisfaction (10 , 12 , 16 , 19 , 20). Some evidence also suggests sex-related differences in response to melatonin, with women exhibiting greater and more rapid reductions in anxiety and pain following oral administration (23). When applied locally to the extraction socket, melatonin (3 mg) has been shown to reduce postoperative pain and swelling and to improve maximum mouth opening, while also increasing the bone turnover marker (26). However, radiographic evaluations revealed no significant long-term differences in bone density compared with placebo (8 , 26). With respect to sleep outcomes, melatonin premedication did not significantly improve first-night postoperative sleep quality following third molar surgery (23).

Despite this background, the present trial did not confirm the hypothesized benefits of melatonin in this setting. Although previous studies have reported reductions in preoperative anxiety with melatonin compared with placebo and anxiolytic efficacy comparable to benzodiazepines (10 , 12 , 19 , 20), our findings did not demonstrate significant effects in patients undergoing mandibular third molar extraction. This lack of effect persisted despite the use of a 15 mg dose, which is higher than that commonly reported in the existing literature. Likewise, psychomotor performance assessed by the DSST did not differ between groups. Regarding secondary outcomes, postoperative sleep quality was generally satisfactory in both groups, suggesting that a single preoperative dose of melatonin had minimal influence on subjective sleep during the immediate recovery period. This observation is consistent with previous findings in the context of third molar surgery, which similarly reported no significant differences between melatonin and placebo for early postoperative sleep quality (23).

Third molar removal is frequently associated with psychological stress and, in some cases, may contribute to the development of post-traumatic stress disorder (PTSD)-related symptoms (1). Although the overall psychological burden is often modest, a subset of patients may experience clinically meaningful distress. Previous studies have reported that up to 27% of patients present Impact of Event Scale-Revised (IES-R) scores suggestive of clinical concern or probable PTSD one week after surgery (1), while 4-8% meet thresholds indicative of PTSD at one month postoperatively (24 , 25). Increased vulnerability has been associated with factors such as greater intraoperative pain (24), longer surgical duration (25), and a history of distressing dental experiences (1 , 24). In the present study, PTSD-related symptoms were identified in a small proportion of patients (4%) during the first postoperative week, a prevalence consistent with previously reported ranges (24 , 25). This relatively low frequency may be explained by the study design, which excluded very difficult cases based on the Kharma classification, involved relatively short surgical times, and was performed by experienced surgeons-factors likely to have minimized procedural distress. Within this clinical context, preoperative melatonin administration did not demonstrate a protective effect against early PTSD-related symptoms.

Beyond third molar extraction, melatonin has demonstrated potential across multiple fields of dentistry due to its anti-inflammatory, antioxidant, immunomodulatory, analgesic, and bone-remodeling properties (6 - 9 , 14 , 15). In periodontology, both systemic and topical melatonin (1-10 mg), when used as an adjunct to nonsurgical periodontal therapy, have been associated with improvements in probing depth, clinical attachment level, and bone regeneration, along with reductions in inflammatory mediators such as interleukin-6 and tumor necrosis factor- (7 , 14 , 27). In oral surgery, melatonin has also exhibited osteogenic potential in settings such as mandibular distraction osteogenesis and post-extraction healing, with some studies reporting enhanced bone formation (6 , 8 , 15 , 17). In implant dentistry, systemic and local melatonin administration has been linked to improved osseointegration and peri-implant bone regeneration (6 , 14). Additionally, evidence suggests that melatonin may act as an effective preemptive analgesic in periodontal flap surgeries, providing postoperative pain control comparable to nonsteroidal anti-inflammatory drugs while maintaining a favorable safety profile (9).

Study limitations

This study presents limitations that should be considered when interpreting the findings. First, baseline imbalances in anxiety and psychomotor scores limited the original plan for repeated-measures ANOVA and required the use of ANCOVA and GLM, which may have reduced statistical power. Second, the sample consisted of young, systemically healthy individuals (ASA I-II) undergoing procedures of moderate difficulty according to the Kharma classification, which limits the generalizability of the findings to older patients, those with higher anxiety, or individuals with comorbidities. Third, only a single fixed 15 mg sublingual dose of melatonin was administered; this regimen may not represent the optimal dosing strategy, as neither dose-response effects, pharmacokinetics, nor repeated dosing were assessed. Fourth, the assessment of sleep quality relied on a simple, non-validated three-category patient report, which may lack sensitivity compared to standardized measures. Finally, PTSD assessment was limited to one week postoperatively, capturing only early psychological responses rather than long-term sequelae.

## Conclusions

Within the specific setting of mandibular third molar extraction, the present findings indicate that a single 15 mg preoperative dose of sublingual melatonin offers no significant benefit over placebo for reducing perioperative anxiety, influence psychomotor function, enhancing sleep quality, or preventing early post-traumatic stress symptoms.

## Figures and Tables

**Table 1:** Table Baseline characteristics and surgical difficulty of mandibular third molars according to the kharma classification, by treatment group.

Tooth characteristics	N	Melatonin	Placebo	Test Statistic
		(N=24)	(N=24)	
Position (Kharma)	48			Î§2=1.46, P=0.69
Mesioangular		5/24	7/24	
Horizontal		4/24	6/24	
Vertical		13/24	9/24	
Distoangular		2/24	2/24	
Ramus Relationship/ Space Available (Kharma)	48			Î§2=2.81, P=0.25
Class 1		18/24	13/24	
Class 2		6/24	10/24	
Class 3		0/24	1/24	
Depth (Kharma)	48			Î§2=1.53, P=0.46
Level A		0/24	1/24	
Level B		15/24	12/24	
Level C		9/24	11/24	
Roots Form (Kharma)	48			Î§2=1.62, P=0.44
Convergent		15/24	19/24	
Divergent		7/24	4/24	
Bulbous		2/24	1/24	
Unfavorable Roots		6/24	4/24	

N is the number of non-missing value. Χ2= Chi-Square Test.

**Table 2:** Table Raw (unadjusted) state anxiety (stai-s and vas-a) and trait anxiety (stai-t) scores at baseline and after drug administration, by treatment group (higher scores indicate greater anxiety).

Anxiety Measurements	Melatoninmean (SD)	Placebomean (SD)	Total	p-value
STAI-T single measure	43.0 (6.6)	40.2 (6.7)	41.6 (6.8)	0.15
STAI-S measure 1 (baseline)	41.6 (8.3)	36.2 (8.9)	38.9 (9.0)	0.03
STAI-S measure 2 (45 minutes after medication)	38.4 (8.9)	33.2 (7.6)	35.8 (8.6)	0.03
STAI-S measure 3 (after surgery)	38.0 (7.9)	33.1 (9.4)	35.5 (8.9)	0.06
VAS-A measure 1 (baseline)	42.8 (20.7)	27.4 (22.2)	35.1 (22.6)	0.01
VAS-A measure 2 (45 minutes after medication)	30.5 (23.3)	21.2 (21.1)	25.9 (22.5)	0.15
VAS-A measure 3 (after surgery)	20.0 (19.4)	12.5 (19.6)	16.2 (19.7)	0.19

P-value is based in Student test, with assumptions based on Levene and Shapiro-Wilk tests. STAI, State-Trait Anxiety Inventory. STAI-S, state anxiety. STAI-T, trait anxiety. VAS-A, Visual Analog Scales for anxiety.

**Table 3:** Table Surgeon-assessed intraoperative patient anxiety and behavioral responses measured by the interval scale of anxiety response (ISAR), by treatment group (higher ISAR indicates greater anxiety).

	Interval Scale of Anxiety Response (ISAR)(Scale 0-10)	Melatonin (Mean SD)	Placebo (Mean SD)	Total(Mean SD)	p-value
1	Patient perspired more than appropriate for the room temperature	0.8 (1.9)	1.1 (2.3)	1.0 (2.1)	0.52
2	Patient showed increased muscle tension (e.g., white-knuckling/gripping the chair)	2.5 (3.4)	2.8 (3.0)	2.6 (3.2)	0.43
3	Patient's respiratory rate increased	0.7 (1.6)	0.5 (1.4)	0.6 (1.5)	0.70
4	Patient displayed nervous tremors	1.0 (2.0)	1.5 (3.1)	1.2 (2.6)	0.70
5	Patient displayed facial signs of anxiety (grimacing, wide eyes, tightly closed eyes, pallor)	1.6 (2.4)	2.9 (3.3)	2.2 (2.9)	0.17
6	Patient displayed vocal signs of anxiety (crying, moaning, gasping, trembling voice)	1.5 (2.9)	1.2 (3.0)	1.4 (2.9)	0.56
7	Patient verbally reported feeling anxious, nervous, or scared	2.0 (3.2)	2.8 (3.5)	2.4 (3.4)	0.42
8	Patient asked anxiety-related questions (e.g., about the procedure's steps, pain, necessity)	0 (0.0)	1 (4.2)	1 (2.1)	0.34
9	Patient interrupted the procedure (e.g., requested a pause, placed hands near mouth, refused to open mouth)	0.4 (1.2)	1.6 (3.3)	1.0 (2.6)	0.32
10	Overall, I felt the patient was anxious	2.8 (2.9)	3.0 (3.4)	2.9 (3.1)	0.91
11	To what degree did the patient's anxiety or agitation interfere with the procedure?	1.2 (2.5)	1.6 (2.5)	1.4 (2.5)	0.30
	ISAR total sum of scores	14.5 (19.2)	19.5 (26.2)	17.0 (22.9)	0.63

P-values are based on the Mann-Whitney U test, which was applied due to violations of homogeneity of variance (Levene's test) and normality (Shapiro-Wilk test).

**Table 4:** Table Scores on the impact of event scale-revised (IES-R) subscales and total score assessing post-traumatic stress symptoms one week after mandibular third molar surgery, by treatment group (higher IES-R indicates greater distress).

Impact of Event Scale-Revised (IES-R)	Melatonin Mean (SD)	Placebo Mean (SD)	Total Mean (SD)	p-value
IES-R Intrusion	1.7 (2.2)	2.5 (3.1)	2.1 (2.7)	0.36
IES-R Avoidance	1.9 (3.0)	2.2 (4.4)	2.0 (3.7)	0.58
IES-R Hyperarousal	1.8 (1.8)	2.3 (2.9)	2.0 (2.4)	0.82
IES-R Total	5.3 (5.6)	7.0 (10.0)	6.1 (8.1)	0.90

P-values are based on the Mann-Whitney U test, which was applied due to non-normal data distribution (Shapiro-Wilk test) and heterogeneity of variance (Levene's test).

## Data Availability

Declared none.
